# Level-Set Interface Description Approach for Thermal Phase Change of Nanofluids

**DOI:** 10.3390/nano12132228

**Published:** 2022-06-29

**Authors:** Ali Yahyaee, Amir Sajjad Bahman, Klaus Olesen, Henrik Sørensen

**Affiliations:** 1 Department of Energy, Aalborg University, 9220 Aalborg, Denmark; hs@energy.aau.dk; 2 Danfoss Silicon Power, 24941 Flensburg, Germany; klaus_olesen@danfoss.com

**Keywords:** thermal phase change, nanofluids, interface curvature, level-set, volume of fluid, benchmark study

## Abstract

Simulations of thermally driven phase change phenomena of nanofluids are still in their infancy. Locating the gas–liquid interface location as precisely as possible is one of the primary problems in simulating such flows. The VOF method is the most applied interface description method in commercial and open-source CFD software to simulate nanofluids’ thermal phase change. Using the VOF method directs to inaccurate curvature calculation, which drives artificial flows (numerical non-physical velocities), especially in the vicinity of the gas–liquid interface. To recover accuracy in simulation results by VOF, a solver coupling VOF with the level-set interface description method can be used, in which the VOF is employed to capture the interface since it is a mass conserving method and the level-set is employed to calculate the curvature and physical quantities near the interface. We implemented the aforementioned coupled level-set and VOF (CLSVOF) method within the open-source OpenFOAM${}^{®}$ framework and conducted a comparative analysis between CLSVOF and VOF (the default interface capturing method) to demonstrate the CLSVOF method’s advantages and disadvantages in various phase change scenarios. Using experimental mathematical correlations from the literature, we consider the effect of nanoparticles on the base fluid. Results shows that the new inferred technique provides more precise curvature calculation and greater agreement between simulated and analytical/benchmark solutions, but at the expense of processing time.

## 1. Introduction

Recently, there has been a rising interest in the application of thermal phase change processes to nanofluids, particularly flow boiling of nanofluids [1,2,3]. Flow boiling is an efficient cooling technique because it removes latent heat of vaporization while pushing the flow owing to the density difference. Furthermore, nanofluids can be used to improve heat transfer in industrial applications, which was proved to be a very successful technique by Choi and Eastman [4] for the first time. Following that, several researchers began to investigate various aspects of employing nanofluids in cooling applications both experimentally [5,6,7,8,9,10,11] and numerically [12,13,14,15,16,17,18,19].

Phase change research was generally experimental; however, experiments in this field are restricted, and even a little mistake might result in a huge error [20]. As a result, it is necessary to numerically model phase change processes in order to fully comprehend the available experimental data and to accurately characterize the underlying physics. The ability to examine complex geometries and various conditions is one of the most important benefits of numerical simulation [21,22].

**Figure 1:**
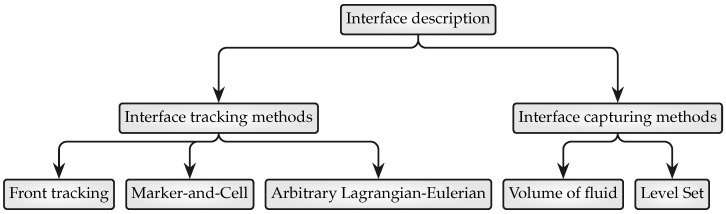
An overview of the numerical approaches and their branches that have been introduced and implemented for the description of the dispersed phase topology or for the localization of the interface between two phases.

While phase transition has been researched for more than half a century, its complicated physical nature, which involves simultaneous heat, mass, and momentum transfer, makes numerical predictions difficult. Locating the interface position is a significant difficulty in CFD modeling of two-phase flows. The issue is how to construct a sharp interface by analyzing the curvature of the interface and forecasting physical parameters that change dramatically across the interface. As seen in Figure 1, the gas–liquid interface can be represented using interface tracking and capturing techniques. The interface tracking techniques are primarily divided into three categories: front tracking methods, marker methods, and arbitrary Lagrangian–Eulerian methods. The primary advantage of this technique is its ability to appropriately analyze the interface profile; however, since the computational domain requires meshing updates when the interface deforms, it will be prohibitively difficult to precisely monitor the interface while maintaining a reasonable degree of re-meshing repetition. This is related to the production of an overwhelming mesh, which renders interface tracking approaches outdated.

As an alternative to interface tracking techniques, interface capturing techniques can be discussed, which specify the interface location using a scalar function. The volume-of-fluid (VOF) and level-set (LS) functions are the most well-known scalar functions for describing the gas–liquid interface. The VOF method, which was pioneered by [23], has evolved into a norm for both open-source and commercial CFD software. A volume fraction (α) is employed in the VOF technique to distinguish between the fluid 1 and fluid 2 inside the domain. The cell is filled with fluid 1 when α=1, and with fluid 2 when α=0. Each phase’s volume fraction is tracked across the domain in this model, which uses a single momentum equation to address the fluids.

OpenFOAM’s phase change solvers mostly employ VOF to describe interfaces, as well as research into simulating flow boiling of nanofluids, and it has been applied in a broad variety of phase change circumstances, some of which are listed in the Table 1. The volume fraction gradient is used as the normal vector field perpendicular to the gas–liquid interface in the VOF technique. Because the volume fraction is not a smooth function, the direction of the normal vector is chaotic. The curvature of the unit normal vector is equal to its divergence. Additionally, due to the smeared interface, a finite gradient of curvature develops in the normal direction. These impacts lead to an incorrect curvature calculation, which results in a larger numerical error for phase change calculations [24].

Using other interface capture techniques, most notably the level-set approach, results in a more accurate estimation of interface curvature. The level-set approach, presented by [30], calculates a distance function for the entire computational domain’s cells. The zero distance denotes the interface between gases and liquids. The cells with a positive distance are believed to be in the continuous phase (liquid), whereas those with a negative distance are thought to be in the scattered phase (gas). Dhir’s group has extensively employed the level-set technique [31,32,33,34] to investigate a variety of phase change events, including nucleate boiling, film boiling from a horizontal cylinder, and subcooled pool boiling. Apart from Dhir’s group, ref. [35] investigated film boiling using the level-set approach, and [36] simulated the evaporation of a moving and deforming droplet. The level-set technique, on the other hand, is a non-conservative methodology that does not ensure mass conservation in phase transition calculations [37]. The primary drawback of the level-set approach is the fluid mass loss. Ref. [38] presented a combination approach of level-set and VOF termed CLSVOF, which was extensively adopted by others [39,40,41,42]. It generates a precise, sharp interface and compensates for the level-set method’s drawback. The volume fractions of liquid and gas are calculated using VOF, and level-set determines geometrical interface properties such as the curvature and normal vector in each cell.

According to the best knowledge of the writers, prior research on nanofluid flow boiling has used VOF to capture the interface; however, this technique produces less accurate gas–liquid curvature calculations, which in turn yields in less accurate results. Due to the fact that numerous studies in the literature have identified level-set as an interface description method for calculating curvature, level-set is used for the first time in this paper for nanofluids’ thermally driven phase change modeling, and the obtained results are compared to VOF’s utilizing benchmark scenarios to demonstrate their respective strengths and limitations in different thermal phase change circumstances. Benchmark instances are

- Evaporation by heat conduction,
- Condensation by heat conduction,
- Film condensation on a vertical plate,
- 2D film boiling.

We picked these four benchmark cases because of their relevance in a wide range of processes: one-dimensional boiling, one-dimensional condensation, two-dimensional condensation, and two-dimensional boiling. To ensure the solver’s accuracy, simulation results were validated using analytical solutions.

## 2. Numerical Formulation

The mass, momentum, and energy conservation equations as well as the advection interface description equations are expressed as follows for two incompressible and immersible fluids

(1) $$\frac{\partial \rho }{\partial t}+\nabla \cdot \left(\rho U\right)=0,$$

(2) $$\frac{\partial (\rho U)}{\partial t}+\nabla \cdot \left(\rho UU\right)=-\nabla p+\nabla \cdot \tau +\rho g+F,$$

(3) $$\frac{\partial \alpha }{\partial t}+U\cdot \nabla \alpha =-m^{{}^{\prime \prime }}\left[\frac{1}{\rho_{l}}-\alpha \left(\frac{1}{\rho_{l}}-\frac{1}{\rho_{v}}\right)\right],$$

(4) $$\frac{\partial (\rho c_{p}T)}{\partial t}+\nabla \cdot \left(\rho c_{p}UT\right)=\nabla \cdot \left(k\nabla T\right)-m^{{}^{\prime \prime \prime }}\left(h_{v}-h_{l}\right),$$

where $m^{{}^{\prime \prime \prime }}$ represents the rate of exchanged volumetric mass flux, which was determined by applying the Tanasawa model ([43]). In this model, the interface superheat temperature (ΔTsat) is used to determine the interface mass flux:

(5) $$m^{{}^{\prime \prime \prime }}=\frac{2\gamma }{2-\gamma }{\left(\frac{M_{l}}{2ßR}\right)}^{1/2}\frac{\rho_{v}\left(h_{v}-h_{l}\right)\Delta T_{\mathrm{sat}}\left|\nabla \alpha \right|}{T_{\mathrm{sat}}^{3/2}},$$

where γ is the evaporation coefficient (here ir is equal to 1), $M_{l}$ is the fluid’s molecular weight, and *R* the universal gas constant.

The domain is created for a mixture, using the α function to differentiate the liquid and gas. In this study, the interface description is provided via the use of two separate techniques for interface capture, VOF and CLSVOF. The VOF approach represents the interface and separate phases using a liquid (nanofluid) volume fraction function (α).

(6) $$\alpha =\frac{V_{\mathrm{nf}}}{V}=\left\{\begin{array}{cc} 1 & \mathrm{nanofluid}\;\\ 0<\alpha <1 & \mathrm{Interface}\;\\ 0 & \mathrm{Vapor}\; \end{array}\right.,$$

where α denotes the nanofluid’s volume fraction, $V_{\mathrm{nf}}$ denotes the volume of nanofluid contained inside a cell, and *V* is the total cell volume. The volume fraction function of liquid (α) is also used to determine the density of a mixture ($\rho_{m}$), the viscosity of a mixture ($\mu_{m}$), the constant pressure specific heat of a mixture ($c_{p,m}$), and the thermal conductivity of a mixture ($k_{m}$).

(7) $$\rho_{m}=\alpha \rho_{\mathrm{nf}}+\left(1-\alpha \right)\rho_{v},$$

(8) $$\mu_{m}=\alpha \mu_{\mathrm{nf}}+\left(1-\alpha \right)\mu_{v},$$

(9) $$c_{p,m}=\alpha c_{p,\mathrm{nf}}+\left(1-\alpha \right)c_{p,v},$$

(10) $$k_{m}=\alpha k_{\mathrm{nf}}+\left(1-\alpha \right)k_{v}.$$

We integrated the CLSVOF technique with VOF into an OpenFOAM solver for thermally induced phase changes. The CLSVOF method makes use of the VOF method’s mass conservatism and the LS method’s interface sharpness. A new scalar field termed level-set function (ψ) is defined in the CLSVOF method instead of α, which is used in VOF method. The reader is directed to Omar et al’s [24] paper for more thorough details on the CLSVOF method’s implementation and governing equations.

### 2.1. Governing Equations Used to Calculate Nanofluids Thermophysical Properties

This paper investigates nanofluids thermally driven phase change cases. Nanofluids are assumed to be homogeneous, and their properties can be determined experimentally or theoretically. The density, specific heat capacity, viscosity, thermal conductivity, and surface tension of a nanofluid are calculated using [44,45,46,47]:

(11) $$\rho_{\mathrm{nf}}=\phi_{\mathrm{np}}\rho_{\mathrm{np}}+\left(1-\phi_{\mathrm{np}}\right)\rho_{\mathrm{bf}},$$

(12) $$\mu_{\mathrm{nf}}=\left(1+2.5\phi_{\mathrm{np}}\right)\mu_{\mathrm{bf}},$$

(13) $$c_{p,\mathrm{nf}}=\frac{\phi_{\mathrm{np}}\rho_{\mathrm{np}}c_{p,\mathrm{np}}+\left(1-\phi_{\mathrm{np}}\right)\rho_{\mathrm{bf}}c_{p,\mathrm{bf}}}{\rho_{\mathrm{nf}}},$$

(14) $$k_{\mathrm{nf}}=\frac{k_{\mathrm{np}}+\left(n-1\right)k_{\mathrm{bf}}-\left(n-1\right)\phi_{\mathrm{np}}\left(k_{\mathrm{bf}}-k_{\mathrm{np}}\right)}{k_{\mathrm{np}}+\left(n-1\right)k_{\mathrm{bf}}+\phi_{\mathrm{np}}\left(k_{\mathrm{bf}}-k_{\mathrm{np}}\right)},$$

(15) $$\frac{\sigma_{\mathrm{bf}}-\sigma_{\mathrm{nf}}}{\sigma_{\mathrm{bf}}}=b\;\ln\left(\frac{\phi_{\mathrm{np}}}{a}+1\right),$$

where $\phi_{\mathrm{np}}$ is the volumetric concentration of nanoparticles. The number *n* specifies the empirical shape factor, which is equal to three for spherical nanoparticles. The np, bf, and nf, respectively, indicate the characteristics of nanoparticles, base fluid, and obtained nanofluid. The experimental values (a and b) are $7.673\times 10^{-7}$ and $-7.773\times 10^{-3}$[47]. Spherical Al_2_O_3_ nanoparticles with $\phi_{\mathrm{np}}=0.01$ are added to the base fluid.

### 2.2. Discretization Schemes for Solution Algorithm

OpenFOAM has a number of built-in numerical schemes for discretizing the terms in each conservation equation. As seen in Table 2, second-order schemes are used in different cases for all OpenFOAM calculations in this study.

## 3. Benchmark Study

The VOF and CLSVOF methods in thermal phase change cases are compared in this section. Experimental correlations and analytical solutions serve as the fundamental comparison ground for quantifying the results of different benchmark situations. To quantify the outcomes for various benchmark scenarios, analytical solutions and experimental correlations were employed as the basic comparison ground. The Latex routines were used to generate schematics of benchmark cases and graphs illustrating the findings. It is critical to note that when we refer to liquids in the following benchmark situations, we are referring to nanofluidic liquids.

The study was conducted using different benchmark cases and a systematic grid refinement. All the benchmark cases were created using simple rectangular blocks. These blocks are divided into $N_{k}$ grids. For example, $N_{k}=\left[30,80,130,180\right]$ are the number of grids in the first two benchmark cases. If $e_{k}$ is the error generated by dividing the domain into $N_{k}$ grids, convergence rate ($R_{k}$) is defined as

(16) $$R_{k}=\frac{\log(e_{k}/e_{k-1})}{\log(N_{k}/N_{k-1})}.$$

### 3.1. Evaporation by Heat Conduction

**Figure 2:**
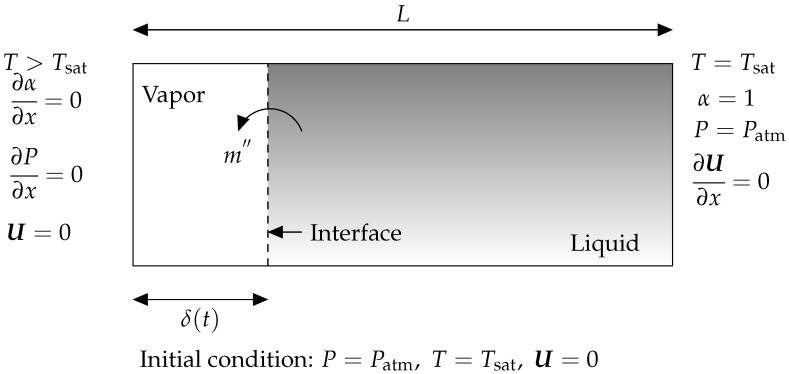
The graphic depicts the 1D Evaporation by heat conduction problem (also known as the Stefan problem) and the boundary conditions associated with it. Gray denotes liquid, while white denotes a part of geometry that is filled with vapor at a saturated temperature. The phase change occurs at the gas–liquid interface, shown by the dashed line. Phase change is induced when the wall in contact with the vapor reaches a temperature greater than superheat. As a result of the evaporation phase change previously described, the interface, which also serves as a measure for the vapor layer thickness, is shifting to the right.

The Stefan problem, also known as evaporation by heat conduction, is a 1D benchmark example that is generally utilized to test thermally driven phase change mass transfer in a new solver. Figure 2 illustrates the 1D evaporation by heat conduction problem schematically. Without convection, the left wall’s greater temperature induces evaporation at the interface, resulting in interface motion to the right. Evaporation is entirely controlled by conduction heat transfer. Table 3 contains the thermophysical characteristics utilized to solve this test case.

The analytical solutions [48] for the temperature distribution (T(x,t)) and the thickness of vapor film (δ) are as follows

(17) $$T\left(x,t\right)=T{|}_{x=0}-\frac{\Delta T_{\sup}}{\mathrm{erf}(\epsilon )}\;\mathrm{erf}\left(\frac{x}{2\sqrt{\frac{k_{v}t}{\rho_{v}c_{p,v}}}}\right),$$

(18) $$\delta \left(t\right)=2\epsilon \sqrt{\frac{k_{v}t}{\rho_{v}c_{p,v}}},$$

where ϵ is a constant given by

(19) $$\epsilon \;\exp\left(\epsilon^{2}\right)\;\mathrm{erf}\left(\epsilon \right)=\frac{c_{p,v}\Delta T_{\sup}}{\left(h_{v}-h_{l}\right)\sqrt{ß}}.$$

**Figure 3:**
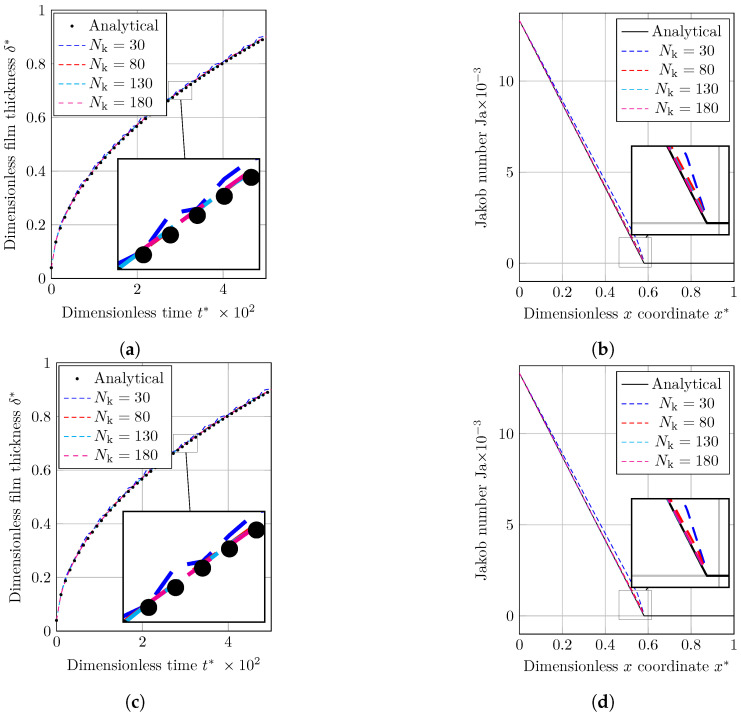
A comparison between the numerical data achieved using the VOF and CLSVOF techniques with the numerical solutions to the evaporation by heat conduction problem. (**a**) The thickening of the vapor film’s dimensionless thickness with respect to time as calculated using the VOF technique is shown. (**b**) The Jakob number profile throughout the dimensionless length as determined using the VOF approach is shown. (**c**) The thickening of the vapor film’s dimensionless thickness with respect to time, as determined using the CLSVOF technique. (**d**) CLSVOF technique was used to obtain Jakob number distribution throughout the dimensionless length.

Evaporation by heat conduction benchmark case is solved for $N_{k}=[30,80,$130,180] number of nodes and results for three implemented interface description methods are presented in Figure 3 as

- The dimensionless location of the interface ($\delta^{*}$) through dimensionless time ($t^{*}$), and
- Jakob number distribution (Ja) within the 1D domain to study temperature distribution.

The interface location and time are normalized by scaling with the domain length (*L*) and the Capillary time scale ($t_{\sigma }$) which itself is

(20) $$t_{\sigma }=\sqrt{\frac{\rho_{v}L^{3}}{\sigma }}.$$

The Jakob number, here, is introduced to present a dimensionless display of temperature profile and is presented as

(21) $$\mathrm{Ja}=\frac{c_{p,v}\left(T-T_{\mathrm{sat}}\right)}{\left(h_{v}-h_{l}\right)}.$$

As shown in Figure 3a,c, when we use the coarsest grid structure ($N_{k}=30$) the dimensionless vapor film thickness ($\delta^{*}$) graph has a zigzag path. When we use more refined grid structures, this zigzag path is gradually turned into curved lines (noises are dumped), and results become closer the analytical solution.

The Jakob number along domain at t=20 s is shown in Figure 3b,d. As can be seen, the Jakob number close to the gas–liquid interface shows higher values than the analytical solution, in particular when coarser grid structures ($N_{k}=\left[30,80\right]$) are used for the simulation. This is due to the fact that a source term implicitly sets the interface temperature to saturation [49,50].

By looking at Figure 3b,d, we cannot uncover how different implemented interface description methods are functioning compared to each other. So to reach this goal, we study the logarithmic error graph for different methods. The L2 error norm is used to quantify the aforementioned difference between simulation and analysis. The following equation can be used to obtain the L2 norm of the temperature distribution error ($e_{T}$) for various grid sizes as

(22) $$e_{T}=\sqrt{\frac{\sum_{i=1}^{N_{k}}{\left(\frac{T_{\mathrm{num}},i-T_{\mathrm{ana}},i}{T_{\mathrm{ana}},i}\right)}^{2}}{N_{k}}}.$$

**Figure 4:**
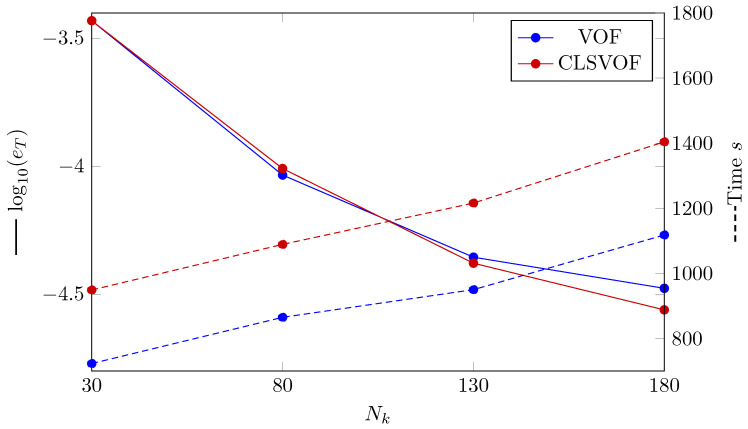
(Solid lines) The error’s logarithmic representation where $e_{T}$ is the L2 norm error computed by Equation (22). (Dashed lines) VOF and CLSVOF computation times for the Stefan problem at various grid sizes.

The L2 norm error of the temperature profile is shown in Figure 4. The convergence with using finer grid structure is seen for all VOF and CLSVOF. CLSVOF has a somewhat lower error rate than VOF, as can be observed. According to results in Figure 4, the rate of convergence (calculated using Equation (16)) for two grid structures with finest mesh size ($N_{k}=\left[130,180\right]$) are calculated and states that CLSVOF with the convergence rate of 0.125 has a better performance than VOF with convergence rate of 0.085.

A dashed line shows the analyzed calculation time for the various number of grids in Figure 4, the VOF approach is proved to be quicker for all grid sizes.

### 3.2. Condensation by Conduction

**Figure 5:**
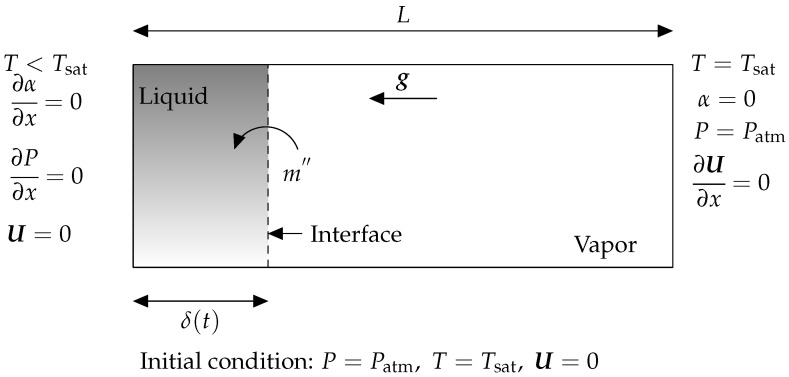
The graphic depicts the 1D condensation by heat conduction problem (also known as the Horizontal film condensation problem) and the accompanying boundary conditions. Gray signifies liquid, whereas white suggests a vapor-filled region of geometry. Both vapor and base liquid are at their saturation temperature. The phase transition takes place at the gas–liquid interface, as shown by the dashed line. Phase change occurs when the wall in contact with the nanofluid cools below superheat. As a consequence of the condensation phase change explained above, the interface, which also acts as a measure for the thickness of the condensed water layer, is moving to the right.

Condensation by conduction, also called Horizontal film condensation problem, is a 1D benchmark case, whose computation results are confirmed with Nusselt’s film theory [51]. The benchmark case schematic with its boundary conditions is shown in Figure 5. In the absence of convection, the lower temperature of left wall causes condensation on this wall, increasing liquid film thickness and causing rightward interface motion. Table 3 contains the thermophysical characteristics utilized in this test scenario.

The analytical solution [48] to calculate the liquid film thickness ($\delta_{\mathrm{an}}\left(t\right)$) is given as

(23) $$\delta_{\mathrm{an}}\left(t\right)={\left[2t\left(\frac{k_{l}}{\rho_{l}c_{p,l}}\right){\left(\frac{1}{2}+\frac{h_{v}-h_{l}}{c_{p,l}\Delta T_{\mathrm{sub}}}\right)}^{-1}\right]}^{\frac{1}{2}}.$$

**Figure 6:**
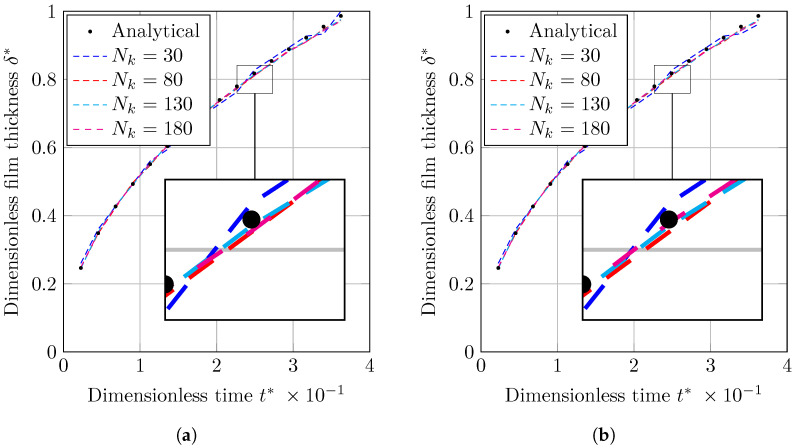
Variation of dimensionless liquid film thickness with dimensionless time achieved by (**a**) VOF and (**b**) CLSVOF for condensation by heat conduction study case.

In this investigation, four grid configurations with $N_{k}$ equal to [30,80,130,180] grids in x direction were used. The results will be liquid film thickness (δ) versus time (*t*), which are made dimensionless by normalizing by *L* and $t_{\sigma }$ (Equation (20)), respectively. Changes in the dimensionless thickness of a condensed liquid layer ($\delta^{*}$) versus dimensionless time ($t^{*}$), obtained using the VOF and CLSVOF methods, are given in Figure 6. As shown, the results match to the analytical results for all mesh sizes except the coarsest one, which is associated with some distances from the analytical solution.

**Figure 7:**
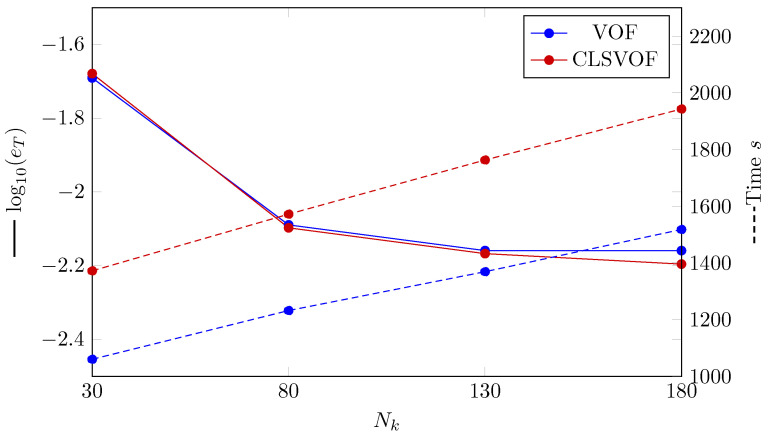
(Solid lines) The error’s logarithmic representation where $e_{\delta }$ is the L2 norm error computed by Equation (24). (Dashed lines) VOF and CLSVOF computation times for the condensation by heat conduction case problem at various grid sizes. The number of nodes along the *x* axis is represented by the horizontal axis ($N_{k}$).

As with the previous benchmark example, the L2 error is calculated for the VOF and CLSVOF techniques (Figure 7). The L2 error for the condensed film thickness is defined as

(24) $$e_{\delta }=\sqrt{\frac{\sum_{i=1}^{N_{\delta t}}{\left(\frac{\delta_{\mathrm{num}},i-\delta_{\mathrm{ana}},i}{\delta_{\mathrm{ana}},i}\right)}^{2}}{N_{\delta t}}},$$

where $N_{\delta t}$ is the number of time intervals. As shown in Figure 7, with grid structures of $N_{k}=\left[30,80,130\right]$ all methods provide similarly precise results. At the finest grid structure ($N_{k}=180$) VOF cannot keep up with CLSVOF and deliver higher error. The rate of convergence (Equation (16)) for the grid structures with finest mesh size ($N_{k}=\left[130,180\right]$) for two methods are calculated and states that CLSVOF with the convergence rate of 0.04 perform better than VOF with convergence rate of $4.8\times 10^{-5}$.

In Figure 7, the investigation on computational time study is demonstrated as dashed lines. As shown in this figure, VOF delivers faster computation compared to CLSVOF.

### 3.3. Film Condensation on a Vertical Plate

**Figure 8:**
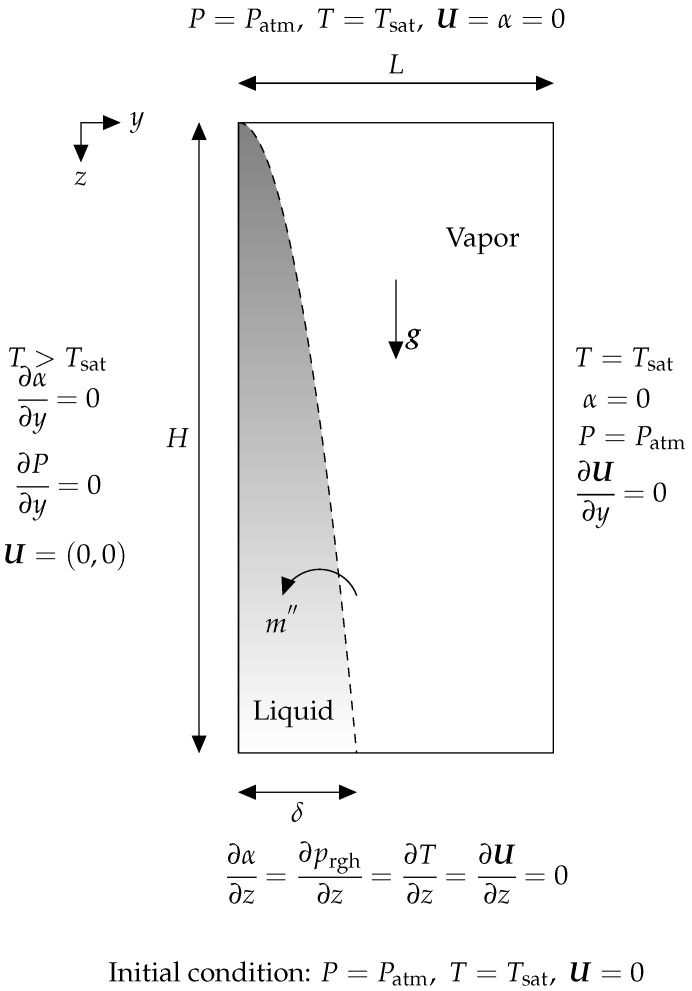
The condensation of a laminar film on a vertical plate is shown schematically, together with the associated boundary conditions. Gray denotes liquid, but white denotes a geometric area filled with vapor. As seen by the dashed line, the phase change occurs at the gas–liquid interface. When the vertical wall cools below superheat, phase change occurs. The condensation phase change described above results in the formation of a layer of condensed liquid in the shape of a parabola on the left wall.

Film condensation on a vertical plate is modeled as a benchmark case and compared with analytical solution. As shown in Figure 8 simulation domain consists of a L×H rectangle in the *y* (normal to the wall) and *z* (along with film flow) directions, which has a nanofluid liquid film with $\delta_{0}$ thickness on its left wall. The left wall temperature is sub-cooled with $\Delta T_{\mathrm{sub}}=20$ K while rest of the domain is filled with saturated vapor ($T_{\mathrm{sat}}=646$ K). Zero gradient condition is used as boundary conditions for α, *T*, U, and *P* at the top and bottom boundaries. The material properties can be seen in Table 4.

To achieve an analytical solution, following assumptions are made [51,52]:

- The temperature profile across the film is linear,
- Effects of forces caused by inertia and interface shearing stress are neglected.

Based on the aforementioned assumptions, the analytical solution [48] for the liquid film thickness (δ) is

(25) $$\delta ={\left[\frac{4\mu_{l}k_{l}\Delta T_{\mathrm{sub}}z}{g\left(h_{v}-h_{l}\right)\rho_{l}\left(\rho_{l}-\rho_{v}\right)}\right]}^{\frac{1}{4}}.$$

Four grid structures of $N_{k}=\left[120\times 240,140\times 280,160\times 320,180\times 360\right]$ are picked for studying this case. A factor of 10 is applied to the grid structure in the *y* and *z* axis to achieve fine mesh distribution in the liquid film region. Scaling with *L* and *H* normalizes the liquid film thickness (δ) and the *z* coordinate, whereas scaling with *L* normalizes the *y* coordinate. Table 5 displays the geometric parameter values for a case study of film condensation on a vertical plate.

**Figure 9:**
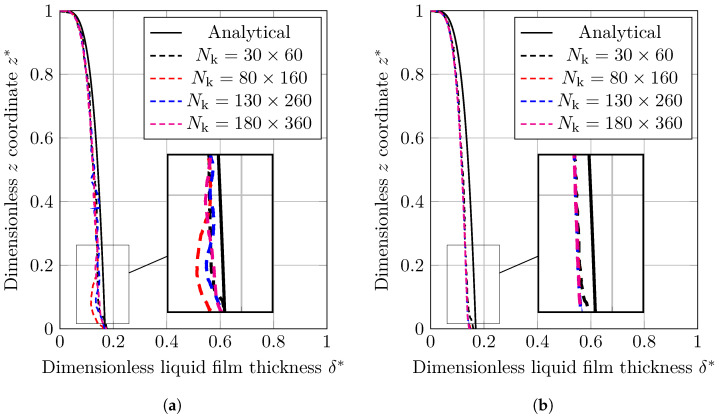
Analytical and numerical results for the normalized thickness of condensed film on a vertical plate predicated using (**a**) VOF and (**b**) CLSVOF.

The dimensionless results for the condensed film thickness using VOF and CLSVOF methods are presented in Figure 9. The results of the VOF simulation are shown to be different from those of the analysis and cannot predict the shape of condensed film correctly, whereas CLSVOF provides more accurate condensed film shape. By refining the grids, the results converge to the analytical solution.

**Figure 10:**
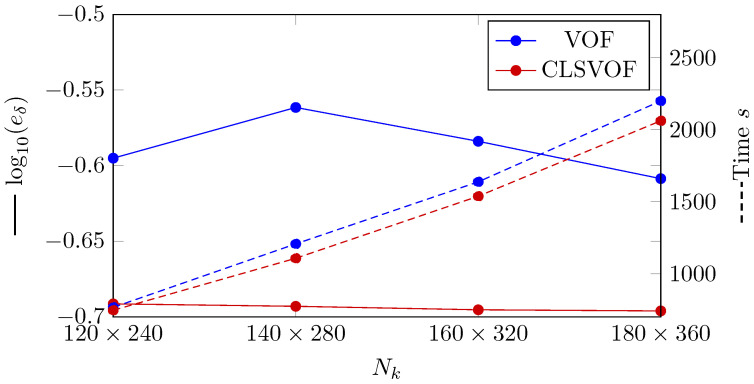
(Solid lines) Logarithmic representation of the error, where $e_{\delta }$ denotes the L2 norm of the error as determined by Equation (24). (Dashed lines) Time required to simulate the vertical film condensation scenario using the VOF and CLSVOF techniques at different grid structures.

To examine the difference between VOF and CLSVOF methods performance, the L2 error calculation is provided in Figure 10. In this case, the L2 error for the condensed nanofluid film thickness is defined by equation

(26) $$e_{\delta }=\sqrt{\frac{\sum_{i=1}^{N_{k}}{\left(\frac{\delta_{\mathrm{num}},i-\delta_{\mathrm{ana}},i}{\delta_{\mathrm{ana}},i}\right)}^{2}}{N_{k}}},$$

where $N_{k}$ is the grid’s number in the *y* axis. As it can be seen in Figure 10, the error generated by CLSVOF is significantly lower than VOF. The rate of convergence (Equation (16)) between the finest mesh structures ($N_{k}=\left[160\times 320,180\times 360\right]$) for CLSVOF method is equal to 0.005, while for VOF has a better convergence with value of 0.17.

In Figure 10 the computational time study is presented too (dashed lines). As shown and unlike previous benchmark cases, CLSVOF method delivers faster computation compared to VOF. This is because VOF struggle to reach the convergence criteria in each time step in this benchmark case.

### 3.4. 2D Film Boiling

**Figure 11:**
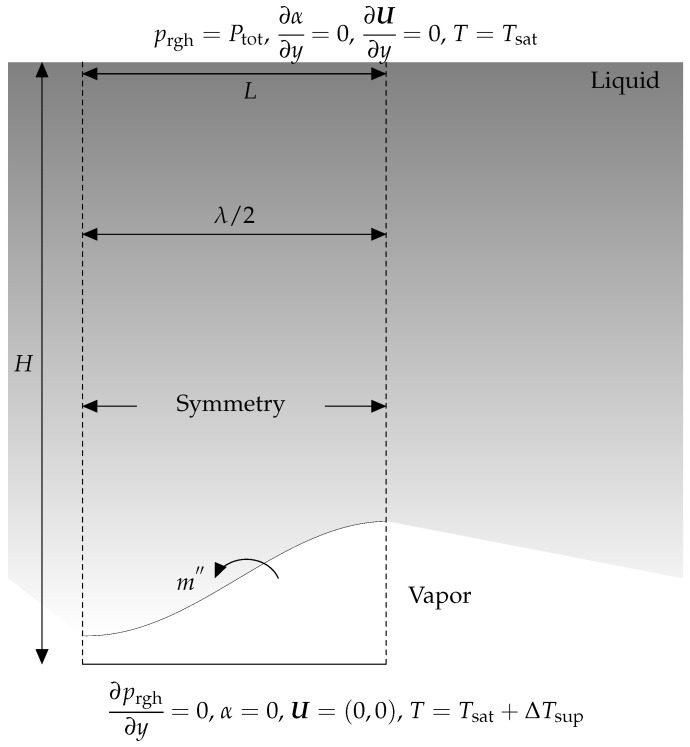
The diagram depicts the 2D film boiling scenario’s geometry and boundary conditions. Between the liquid’s surface and the vapor is a sinusoidal vapor layer. The liquid is at its saturation temperature, but the surface temperature is greater than it. The phase change happens at the liquid–vapor interface, and the bubbles of generated vapor depart the vapor film layer.

The study case is a 2D film boiling problem. As seen in Figure 11, between the surface and the liquid exists a vapor layer in the form of sinusoidal. The liquid is at its saturation temperature ($T_{\mathrm{sat}}=646$ K), but the surface temperature is above it ($\Delta T_{\sup}=5$ K). The phase change happens at the liquid–vapor interface, and the produced vapor is subsequently released as bubbles. The computational domain is defined by the rectangle with the dimension of L=λ/2 and H=λ. In this situation, λ is defined as

(27) $$\lambda =\sqrt{\frac{\sigma }{(\rho_{l}-\rho_{v})g}}.$$

According to Figure 11, the numerical domain exhibits horizontal symmetry. As a result, just a portion of the domain is simulated. Vertical borders are said to have a symmetric boundary condition. The higher border is given the pressure outlet condition, whereas the vertical gradient for other variables is equal to zero.

The thickness of the vapor film is initiated by the following equation,

(28) $$\delta =\frac{\lambda_{0}}{64}\left(4+\cos\left(\frac{2ßx}{\lambda_{0}}\right)\right),$$

where $\lambda_{0}$ is the critical wavelength of Taylor equation, obtained by

(29) $$\lambda_{0}=2ß\sqrt{\frac{3\sigma }{(\rho_{l}-\rho_{v})g}}.$$

The Nusselt number in this case is calculated by

(30) $$\mathrm{Nu}=\frac{\int_{0}^{L}\left(\frac{\lambda }{\Delta T}\frac{\partial T}{\partial y}{|}_{y=0}\right)\;dx}{L}.$$

The outcomes of 2D film boiling scenario can be predicted using a number of empirical relationships. We use the experimental correlation offered by [53]. This correlation define Nusselt number of 2D film boiling as

(31) $$\mathrm{Nu}=0.425\left[\frac{\rho_{v}\left(\rho_{l}-\rho_{v}\right)g\;\left(h_{v}-h_{l}\right)}{k_{v}\mu_{v}\Delta T}\right].$$

Four grid structures of $N_{k}=\left[160\times 320,220\times 440,260\times 520,280\times 560\right]$ are used to analyze these benchmark scenarios. The material properties used to simulate the 2D film boiling benchmark are shown in Table 4. To have the dimensionless numbers as results, *x* and *y* coordinate are normalized by scaling with λ (calculated by Equation (27)) and *t* is become dimensionless by scaling with $t_{\sigma }$ (calculated by Equation (20)).

**Figure 12:**
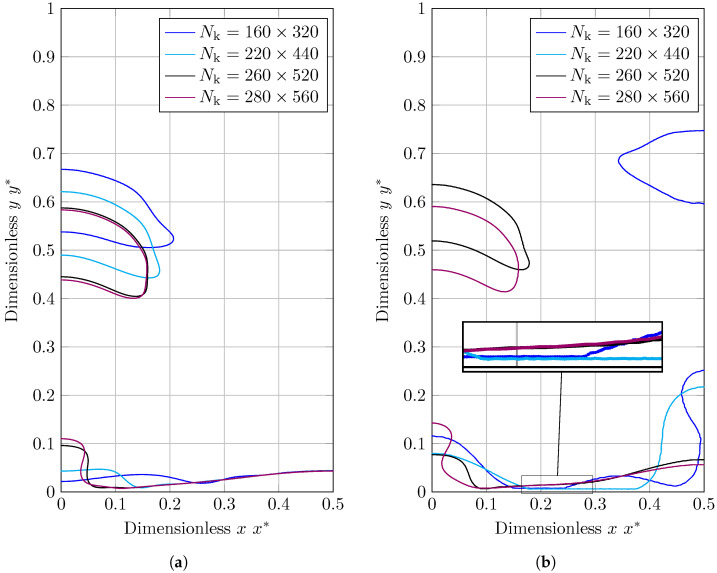
Bubble shape in 2D film boiling benchmark case at a specific height for different grid structures of $N_{k}=\left[160\times 320,220\times 440,260\times 520,280\times 560\right]$). (**a**) VOF; (**b**) CLSVOF.

In Figure 12, the first detached bubble form for VOF and CLSVOF is compared at a given height. The first striking result in Figure 12b is the bubble shape predicted by CLSVOF when the two coarsest grid structures are used ($N_{k}=\left[160\times 320,220\times 440\right]$). The interface is connected to the bottom wall, as can be seen in the magnified image of this graph. This behavior is inconsistent with the benchmark scenario and has not been seen with the VOF and various grid density shown in Figure 12b. This demonstrates that the CLSVOF technique is ineffective when the grid structure is coarse and the interface is close and parallel to the wall. Thus, in the discussion provided for the Nusselt number, the CLSVOF findings for the grid structure $N_{k}=\left[160\times 320,220\times 440\right]$ will be omitted.

VOF and CLSVOF anticipate bubble sizes that are almost identical, but the forms of the bubbles and the curvatures of the bottoms vary. A low-pressure region is formed in the wake of the rising bubbles as a result of the separation and rise of the bubbles. It generates vorticities at the sharp corners of bubbles and affects the bottom curvature.

**Figure 13:**
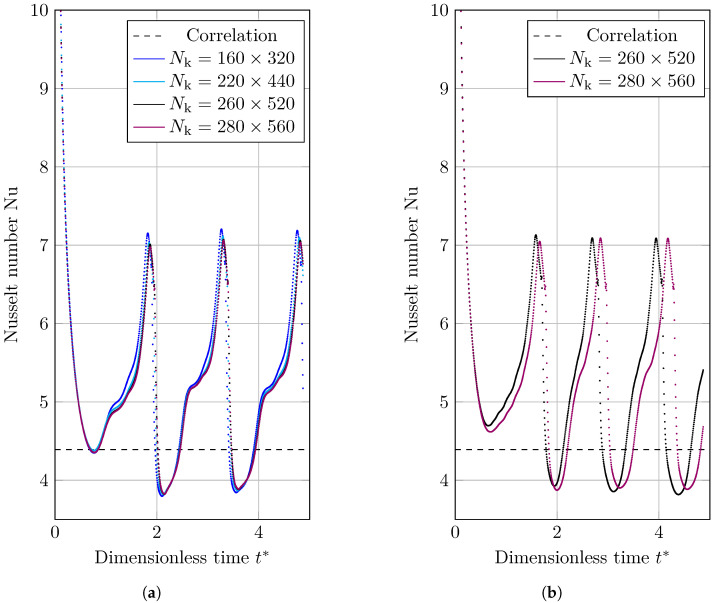
Nusselt number data for the 2D film boiling benchmark scenario that are spatially averaged. The horizontal dashed line is showing the Berenson correlation Nusselt number. (**a**) VOF; (**b**) CLSVOF.

The space-averaged Nusselt number over dimensionless time is shown in Figure 13a,b. The thickness of the film has a significant impact on the Nusselt number. When the vapor layer is thin, the heat flux is larger, and when the film is thick, the heat flux is lower. As the vapor pushes to fill the bubble, the residual layer thins, increasing the average heat flux and Nusselt number; however, following detachment, the vapor returns to the superheated wall and the film thickness increases, leading to a lower Nusselt number.

**Figure 14:**
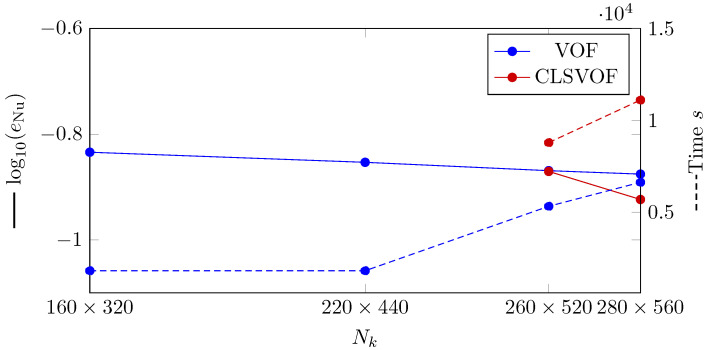
(Solid lines) Logarithmic representation of the error in which $e_{\mathrm{Nu}}$ is the L2 norm of error for Nusselt number calculation. (Dashed lines) Computation time study for 2D film boiling using VOF and CLSVOF techniques at various grid sizes.

In order to perform an individual investigation on the Nusselt number results, the difference between the mean Nusselt number calculated by Berenson correlation (Equation (31)) and time-averaged Nusselt number is presented for VOF and CLSVOF in different studied grid structures (Figure 14). In this case, the error for the time-averaged Nusselt number ($e_{\mathrm{Nu}}$) is defined by equation

(32) $$e_{\mathrm{Nu}}=\frac{\sum_{i=1}^{N_{\delta t}}\frac{{\mathrm{Nu}}_{\mathrm{num},i}-{\mathrm{Nu}}_{\mathrm{cor}}}{{\mathrm{Nu}}_{\mathrm{cor}}}}{N_{\delta t}},$$

where $N_{\delta t}$ is the number of time intervals, ${\mathrm{Nu}}_{\mathrm{num},i}$ mean Nusselt number in the *i*th time step and ${\mathrm{Nu}}_{\mathrm{cor}}$ is the Nusselt number calculated by Berenson correlation. As demonstrated in Figure 14, the error generated by CLSVOF is less than VOF. The rate of convergence (Equation (16)) between the finest grid structures ($N_{k}=\left[260\times 520,280\times 560\right]$) are 0.05 and 0.4 for VOF and CLSVOF, respectively, showing a better convergence for CLSVOF method.

We evaluated the computing time for a bubble separation duration in order to examine the computational time. The bubble detachment period is defined as the time between the detachment of two consecutive bubbles from the vapor film. The computational time study is shown by dashed lines in Figure 10. As can be seen, the VOF method delivers faster computation than CLSVOF for the detachment of one bubble.

## 4. Conclusions

**Figure 15:**
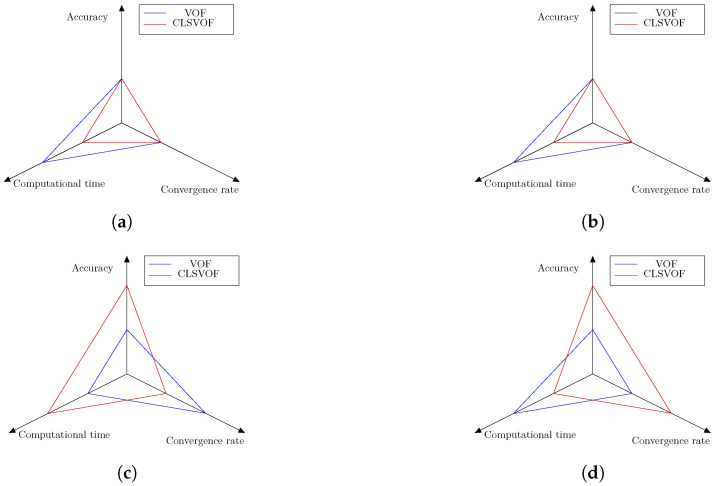
The accuracy, computing time, and convergence rate of the VOF and CLSVOF techniques are shown graphically for four thermally driven phase change benchmark scenarios. Each axis, as indicated, represents a research region and contains two values. If one of the techniques has greater performance in that region, the second value is allocated to that approach, while the first method will receive the first value. (**a**) Stefan problem; (**b**) the condensation by heat conduction; (**c**); 2D vertical film condensation; (**d**) 2D film boiling.

Both OpenFOAM’s default thermally induced phase change solver and prior studies on flow boiling of nanofluids employ VOF to capture the interface; however, this technique calculates less precise gas–liquid curvature, resulting in less accurate results. Given that several studies in the literature have identified level-set as a way for precisely calculating curvature, we combined VOF with level-set (called CLSVOF) and attempted to regain accuracy in simulation results. We have presented quantitative validations of the CLSVOF method versus VOF using a self developed solver. The impact of nanoparticles on the base fluid are considered using empirical equations from the literature. Four scenarios were utilized as test cases. The first test case is the evaporation by heat conduction problem (a one-dimensional evaporation problem); the second is the condensation by heat conduction (also a one-dimensional condensation problem); the third is condensation on a vertical plate (a two-dimensional condensation problem); the fourth is film boiling (a 2D boiling problem). These four test cases were chosen to illustrate a variety of thermal phase transition events. To conclude the benchmark study on these cases, we present Figure 15, which illustrates the performance of the VOF and CLSVOF methods in terms of accuracy, computation time, and convergence rate. As seen in Figure 15, the CLSVOF methodology outperforms or is similar to the VOF method in terms of both accuracy and convergence rate, albeit at the expense of computing time. We can observe that there are no accuracy benefits when simulating 1D benchmark situations with CLSVOF. This is because the curvature in these 1D cases is zero and the improved curvature computation is the basis for CLSVOF’s superior accuracy performance.

## Figures and Tables

**Table 1:** An overview of some of the studies that have used VOF as their interface capture technique is shown in the next section. The scope of these simulations is restricted to thermally induced phase transitions or flow boiling of nanofluids simulations.

Publication	Remarks
Soleimani et al. [25]	The VOF method is used to model subcooled flow boiling of HFE-7100 in a microchannel heat sink with variable concentrations of alumina nanoparticles.
Zhang et al. [26]	In a tiny tube, the VOF approach is used to quantitatively investigate the heat transfer and pressure drop properties of gas–liquid Taylor flows. CuO/water nanofluid was the liquid, while nitrogen was the gas.
Rabiee et al. [27]	flow condensation inside a smooth horizontal tube was simulated. Moreover, the effect of some parameters such as mass flux, tube hydraulic diameter, vapor quality and difference between the wall and saturation temperature on the heat transfer coefficient were investigated.
Yahyaee et al. [28]	This article simulates the development of bubbles and their escape from a superheated horizontal surface. A cylindrical hollow initiates the bubble nucleation process. The simulation will be carried out utilizing VOF using a new OpenFOAM-based two-phase solver.
Abedini et al. [29]	The VOF method are used to explore the subcooled boiling of Alumina-water nanofluid in both vertical concentric annulus and vertical tube. There is a comparison of the inlet vapor volume fraction fluctuation at various nanoparticles concentrations.

**Table 2:** The techniques for discretizing the solution algorithm for benchmark instances are listed in the following table. All of the schemes are standard second-order approaches that can be found in OpenFOAM solvers and are implemented as such.

Terms	Schemes
Temporal term	Backward
Convective term in momentum equation	vanLeerV
Convective term in energy equation	vanLeer
Compression velocity term in momentum equation	interfaceCompression
Diffusion term in momentum equation	Gauss Linear corrected
Viscous term in momentum equation	Gauss Linear

**Table 3:** Base fluid, nanoparticles, and gas properties used in the evaporation by heat conduction (also known as Stefan problem) and condensation by heat conduction (also known as Horizontal film condensation problem) benchmark cases. These two benchmark cases are presented and solved in Section 3.1 and Section 3.2.

	Dimension	Base Fluid	Nanoparticles	Vapor
Thermal conductivity, *k*	$W\;m^{2}\;K^{-1}$	0.648	36	0.03643
Density, ρ	$\mathrm{kg}\;m^{-3}$	645	3600	5.1450
Viscosity, μ	Pa s	$1.48\times 10^{-4}$		$1.502\times 10^{-5}$
Specific heat capacity $c_{p}$	$\mathrm{kJ}\;{\mathrm{kg}}^{-1}\;K^{-1}$	2.794	0.765	2.687
Latent Heat, *h*	$\mathrm{kJ}\;{\mathrm{kg}}^{-1}$	762.52		2777.1
Surface tension, σ	$N\;m^{-1}$	0.045417		

**Table 4:** Base fluid, nanoparticles, and gas properties used in the laminar film condensation and 2D film boiling benchmark instances. These two benchmark cases are presented and solved in Section 3.3 and Section 3.4.

	Dimension	Base Fluid	Nanoparticles	Vapor
Thermal conductivity, λ	$W\;m^{2}\;K^{-1}$	0.531	36	0.538
Density, ρ	$\mathrm{kg}\;m^{-3}$	370.4	3600	242.7
Viscosity, μ	Pa s	$4.53\times 10^{-5}$		$3.23\times 10^{-6}$
Specific heat capacity $c_{p}$	$\mathrm{kJ}\;{\mathrm{kg}}^{-1}\;K^{-1}$	239	0.765	352
Latent Heat, *h*	$\mathrm{kJ}\;{\mathrm{kg}}^{-1}$	1963.5		2240
Surface tension, σ	$N\;m^{-1}$	$7.55\times 10^{-5}$		

**Table 5:** The geometric parameters utilized in the vertical condensation benchmark are shown in the table below. All of the dimensions are shown as functions of the length of the domain. The reader will refer to Figure 8 in order to have a better understanding of this table.

*L*	Length of domain	0.5 L
*H*	Height of domain	3 L
δ(0)	Thickness of condensed film at t=0	0.01 L

## Data Availability

Not applicable.

## Nomenclature

VOF	Volume of Fluid
*t*	Time
***U***	Velocity
*p*	Pressure
g	Gravitational acceleration
F	Surface tension forces
*m*	Transferred mass
*T*	Temperature
*c*	Specific heat
*k*	Thermal conductivity
*R*	Universal gas constant
*h*	Latent heat
*M*	Molecular weight
*V*	Volume of the cell
C	Compressive factor
S	Surface vector
*N*	Number
*e*	error
*R*	convergence rate
Ca	Capillary number
Ja	Jakob number
Nu	Nusselt number
La	Laplace number
$c_{p}$	Dimensionless pressure coefficient
ρ	Density
τ	Deviatoric viscous stress tensor
γ	Evaporation coefficient
μ	Dynamic viscosity
κ	Interface curvature
*p*	constant pressure
l	liquid
v	vapor
sat	saturation
sup	supersaturation
sub	subsaturation
rgh	without the hydrostatic pressure
tot	total
m	mixture
c	compressive
α	volume fraction
σ	capillary
f	cell face
k	nodes in the domain
num	numerical results
ana	analytical results
δ	film thickness
*	dimensionless
${}^{\prime \prime }$	flux rate
